# Vaping for weight control: A cross-sectional population study in England

**DOI:** 10.1016/j.addbeh.2019.04.007

**Published:** 2019-08

**Authors:** Sarah E. Jackson, Jamie Brown, Paul Aveyard, Fiona Dobbie, Isabelle Uny, Robert West, Linda Bauld

**Affiliations:** a Department of Behavioural Science and Health, University College London, UK; b Department of Clinical, Educational and Health Psychology, University College London, UK; c Nuffield Department of Primary Care Health Sciences, University of Oxford, UK; d Usher Institute, College of Medicine and Veterinary Medicine, University of Edinburgh, UK; e Institute for Social Marketing, University of Stirling, UK

**Keywords:** E-cigarettes, Vaping, Weight control, Weight loss, Population survey

## Abstract

**Introduction:**

Concern about weight gain is a barrier to smoking cessation. *E*-cigarettes may help quitters to control their weight through continued exposure to the appetite-suppressant effects of nicotine and behavioural aspects of vaping. This study explored the views and practices of smokers, ex-smokers and current e-cigarette users relating to vaping and weight control.

**Methods:**

Cross-sectional survey of past-year smokers (*n* = 1320), current smokers (*n* = 1240) and current e-cigarette users (*n* = 394) in England, conducted April–July 2018. Data were weighted to match the English population on key sociodemographic characteristics.

**Results:**

Of e-cigarette users, 4.6% (95%CI 2.6–6.6) reported vaping for weight control, and 1.9% (95%CI 0.6–3.2) reported vaping to replace meals/snacks. It was rare for individuals who had smoked in the past year to have heard (8.8%, 95%CI 7.3–10.3) or believe (6.4%, 95%CI 5.1–7.7) that vaping could help control weight. Women (OR = 0.62, 95%CI 0.42–0.93) and older people (OR = 0.30, 95%CI 0.13–0.72) were less likely to have heard the claim and women were less likely to believe it (OR = 0.44, 95%CI 0.27–0.72). However, 13.4% (95%CI 11.3–15.5) and 13.1% (95%CI 11.0–15.2) of current smokers who did not use e-cigarettes said they would be more likely to try e-cigarettes or quit smoking, respectively, if vaping could help control their weight.

**Conclusion:**

One in 16 English people who have smoked in the last year believe that vaping would prevent weight gain after stopping. One in 22 people who vape are using e-cigarettes for this purpose. However, should evidence emerge that e-cigarettes prevent weight gain, one in eight people who smoke would be tempted to quit smoking and use e-cigarettes.

## Highlights

• Concern about weight gain is a barrier to smoking cessation.
• *E*-cigarettes may help quitters to control their weight, but this is yet to be shown.
• Few smokers are aware of a potential link between vaping and weight control.
• If benefits could be demonstrated, smokers may be more inclined quit.

## 1 Introduction

Smoking causes lower body mass index (BMI) (Winsløw, Rode, & Nordestgaard, 2015) probably because of the anorexic effect of nicotine. The behavioural aspects to smoking (hand to mouth action, opening cigarettes, putting cigarettes in the mouth and time taken by smoking) fill time and provide distraction that may influence food consumption (Glover, Breier, & Bauld, 2017) although evidence from low nicotine cigarettes and comparisons between inhalator and patch tend to go against this assumption (West et al., 2001; West, Russell, Jarvis, & Feyerabend, 1984). Smokers are therefore, in general, lighter than non-smokers. As smoking rates have declined in many countries in recent years, obesity rates have risen. One analysis has suggested that up to 14% of the rise in obesity rates in the USA, for instance, could be attributable to the drop in smoking over the same period (Courtemanche, Pinkston, Ruhm, & Wehby, 2016).

There is strong evidence that many people who successfully stop smoking gain weight. A meta-analysis found mean weight gain was 4.8 kg at one year, although there was a lot of variation between individuals, with 13% of quitters gaining >10 kg (Aubin, Farley, Lycett, Lahmek, & Aveyard, 2012). Most weight gain occurred within three months. A prospective cohort study followed individuals to eight years after cessation with mean weight gain of 8.8 kg (Lycett, Munafò, Johnstone, Murphy, & Aveyard, 2011).

Fear of weight gain may be a barrier to smoking cessation. Both adult and younger smokers have reported that they believe smoking helps control weight (Boles & Johnson, 2001; Camp, Klesges, & Relyea, 1993). Girls who are overweight and are concerned about their weight are more likely than those with lower weight concern to start and continue to smoke (French, Perry, Leon, & Fulkerson, 1994; Voorhees, Schreiber, Schumann, Biro, & Crawford, 2002; Wee, Rigotti, Davis, & Phillips, 2001). Fear of weight gain can also be a concern for pregnant smokers and is a barrier to cessation in pregnancy (Bauld et al., 2017; Flemming, McCaughan, Angus, & Graham, 2015). Experience of weight gain may also be a barrier. Some evidence shows that larger weight gain is associated with an increased risk of relapse (Borrelli & Mermelstein, 1998), while other studies report contradictory data (Hall, Tunstall, Vila, & Duffy, 1992).

*E*-cigarettes are currently the most popular aid to stopping smoking in the UK, used in over one third of quit attempts in England (West, Shahab, & Brown, 2016). It has been proposed, but not demonstrated, that e-cigarettes may reduce weight gain on cessation compared with routes to quit without alternative nicotine (Glover et al., 2017). Randomised controlled trials of nicotine replacement therapy (NRT) have shown that stopping smoking with NRT reduces weight gain compared with placebo (Farley, Hajek, Lycett, & Aveyard, 2012). In addition, observational studies that have followed up people who stopped smoking using NRT and who continued to use it in the longer term have found that these people experience less weight gain than those who do not use NRT after stopping smoking. A higher proportion of people who have stopped smoking for at least a year use e-cigarettes than NRT (West & Brown, n.d.). While there is clear evidence from trials of a short-term reduction in weight gain from using nicotine, there is no evidence that short-term use prevents long-term weight gain (Farley et al., 2012). Continued use of a nicotine product may be necessary to benefit. However, other unexplored factors such as the role of flavoured e-liquids as food substitutes, and the behavioural aspects of vaping (e.g. preparing the device, mouth feel), may be important and have not been researched.

To date, there is no strong evidence supporting the hypothesis that vaping prevents weight gain after smoking cessation. However, a few small studies have explored the use of e-cigarettes for weight control. A survey of a convenience sample of adult vapers in the USA who were trying to lose or maintain weight (*n* = 459) (Morean & Wedel, 2017) found that respondents who reported vaping for weight loss or control (13.5% of the sample) were more likely to be overweight already, vaped more frequently, had poor impulse control and preferred particular types of e-cigarette flavours (coffee or vanilla flavour). Another survey of college students in the USA found that while weight concerns were not associated with the use of e-cigarettes per se, greater weight concerns were associated with higher frequency of e-cigarette use (Bennett, Pokhrel, Bennett, & Pokhrel, 2018).

This study aimed to build upon these preliminary findings by assessing the views and practices of current smokers, recent (<1 year) ex-smokers, and current e-cigarette users (overall, exclusive, and dual users) relating to vaping and weight control in a large representative sample of adults in England. Specifically, we aimed to address the following research questions:

1. How prevalent is weight control compared with other motives for smoking and e-cigarette use among i) past-year smokers and ii) current e-cigarette users?
2. How prevalent is the use of e-cigarettes in food-related situations, including snack or meal replacement, compared with other usage?
3. How frequent is, and what motivates, the use of e-cigarettes for snack or meal replacement?
4. What are the beliefs towards using e-cigarettes to prevent weight gain among past-year smokers?
5. In context of e-cigarettes preventing weight gain, what are the attitudes towards quitting and using e-cigarettes among smokers not using e-cigarettes?
6. To what extent are the weight-related motives, usage patterns, beliefs and attitudes, associated with sex, age, ethnicity, social grade, past-year quit attempt and smoking status?

## 2 Method

### 2.1 Study population

The Smoking Toolkit Study is a monthly survey of representative samples of adults in England (Fidler et al., 2011). It monitors trends on a smoking status, consumption, smoking cessation, and use of NRT/e-cigarettes. The study uses a hybrid of random location and simple quota sampling to select a new sample of approximately 1700 adults aged ≥16 years each month. Respondents complete a face-to-face computer-assisted survey with a trained interviewer. Full details of the methods are available elsewhere, and comparisons with national data indicate that key variables such as socio-demographics and smoking prevalence are nationally representative (Fidler et al., 2011).

For the present study, we used aggregated data from respondents to the survey in the period from April to July 2018 as only these waves included items on vaping for weight control. We restricted our analyses to respondents reporting (a) current use of e-cigarettes (either exclusive or dual use) or (b) smoking cigarettes (manufactured or hand-rolled) or any other combustible tobacco product (e.g. pipe, cigar) daily or occasionally in the past year.

### 2.2 Measures

#### 2.2.1 Sociodemographic

Respondents provided data on age, sex, ethnicity (white vs. non-white) and social grade (ABC1, which includes managerial, professional and intermediate occupations, vs. C2DE, which includes small employers and own-account workers, lower supervisory and technical occupations, and semi-routine and routine occupations, never workers and long-term unemployed).

#### 2.2.2 Quitting

Past quit attempts were assessed with the question “*How many serious attempts to stop smoking have you made in the past 12 months? By serious I mean you decided that you would try to make sure you never smoked again*.” This item was coded 0 for smokers who responded that they had not made a quit attempt, and 1 for those who reported one or more quit attempts.

#### 2.2.3 E-cigarette motives, usage patterns, beliefs and attitudes

Use of e-cigarettes was assessed with the item “*Are you using any of the following either to help you stop smoking, to help you cut down or for any other reason at all?*”, with those responding “yes” to the option “electronic cigarette” coded 1 and those responding “no” coded 0. Current e-cigarette users were asked whether they used e-cigarettes for weight control or as a food substitute. Vaping for weight control was assessed with the question “*Which, if any, of the following are important in keeping you using an e-cigarette or vaping device?*”, with those mentioning “it keeps my weight down” coded 1 and those not mentioning weight coded 0. Use of e-cigarettes as a food substitute was assessed with the question “*In which, if any, of the following situations do you regularly use an e-cigarette or vaping device?*”, with those mentioning “to replace a meal or snack” coded 1 and those not mentioning food substitution coded 0. *E*-cigarette users who reported vaping to replace a meal or snack were asked two follow-up questions: 1) “*How often have you used an e-cigarette or vaping device to replace a meal or snack? (never/every day/less than every day but at least once a week/less than once a week but at least once a month/less often than once a month)*” and 2) “*Which, if any, of the following are/were important in using an e-cigarette or vaping device to replace a meal or snack? (flavour/nicotine concentration/keeping my hands busy (e.g. refilling the device)/hand to mouth action/feeling in your mouth/density of vapour/none of these)*”.

Past-year smokers were asked about smoking for weight control and attitudes towards vaping for weight control. Smoking for weight control was assessed with the question “*Which, if any, of the following are [current smokers]/were [ex-smokers] important in keeping you smoking?*”, with those mentioning “it keeps my weight down” coded 1 and those not mentioning weight coded 0. Two items on vaping for weight control asked respondents whether they had ever heard the claim that using an e-cigarette or vaping device can help keep weight down (yes/no), and the extent to which they agreed that vaping can help to keep weight down on a scale from 1 (strongly disagree) to 5 (strongly agree). Responses to the latter question were dichotomised to distinguish those who responded ‘agree’ or ‘strongly agree’ from those who did not agree with the statement.

Current smokers who were not using e-cigarettes were also asked to indicate their agreement with two statements: “*If vaping could help keep my weight down I would be more likely to (i) try e-cigarettes, and (ii) stop smoking*”, on a scale from 1 (strongly disagree) to 5 (strongly agree). For each statement, responses were dichotomised to distinguish those responding ‘agree’ or ‘strongly agree’ from those who did not agree.

### 2.3 Statistical analysis

Our analysis plan was pre-registered on Open Science Framework (https://osf.io/de5br/).

Data were weighted to match the English population profile on age, social grade, region, tenure, ethnicity, and working status within sex. The dimensions are derived monthly from a combination of the English 2011 census, Office for National Statistics mid-year estimates, and an annual random probability survey conducted for the National Readership Survey.

We compared the prevalence of weight control compared with other motives for (i) use of cigarettes among past-year smokers and (ii) use of e-cigarettes among current e-cigarette users. We examined the prevalence of the use of e-cigarettes in food-related situations (e.g. snack or meal replacement) compared with other usage, and the frequency of and motives for use of e-cigarettes as a snack or meal replacement, among current e-cigarette users. We also examined the proportion of past-year smokers who believe vaping can help to keep weight down, and the proportion of current smokers who do not currently use e-cigarettes who agree that they would be more likely to (i) try e-cigarettes and (ii) stop smoking if vaping could help keep their weight down. For each estimate of prevalence, we provide a 95% confidence interval (CI).

We used multivariable logistic regression to examine the extent to which weight control as a motive for cigarette smoking and beliefs and attitudes towards vaping for weight control were associated with sex, age, ethnicity, social grade, past-year quit attempt and smoking status. These factors have previously been shown to be associated with use of e-cigarettes (Brown et al., 2014; Levy et al., 2018; Vardavas, Filippidis, & Agaku, 2015) and obesity/desire for weight control (Dare, Mackay, & Pell, 2015; Martínez, Kearney, Kafatos, Paquet, & Martínez-Gonzélez, 1999; Wardle & Griffith, 2001; Wee et al., 2001; Yaemsiri, Slining, & Agarwal, 2011). Results are presented as adjusted odds ratios (ORs) with 95% CIs. Weight control as a motive for vaping and vaping to replace a meal or snack were so rare that it precluded pre-planned analyses of these variables.

Where results were non-significant, we calculated Bayes factors (BF; planned a priori) to examine whether these associations could best be characterised as evidence of no effect, evidence of an effect, or whether data were insensitive to detect an effect (Dienes, 2014; West, 2015). Alternative hypotheses were represented by half-normal distributions and the expected effect size was set to an odds ratio of 2 based on previous research into sociodemographic variables associated with weight control as a motive for smoking (Fidler & West, 2009). To test sensitivity of the data to detect negative effects, we calculated BFs using the equivalent effect size in the opposite direction (OR = 0.5). In order to calculate BFs using the online calculator specified in our protocol, which only allows expected effects modelled by a half-normal distribution to be expressed positively, we reran each model reversing the categorisation of the relevant outcome variable. A BF ≥ 3 can be interpreted as substantial evidence for the alternative hypothesis (and against the null), while a BF of ≤1/3 can be interpreted as evidence for the null hypothesis. BFs between 1/3 and 3 suggest that the data are insensitive to distinguish the alternative hypothesis from the null (Dienes, 2014; Jeffreys, 1961).

In addition to our pre-planned analyses, we also (i) analysed the prevalence of weight control as a motive for vaping and vaping to replace a meal or snack separately for exclusive and dual users of e-cigarettes, and (ii) reran the logistic regression models on unweighted data to check that the weighting did not substantially alter the results.

All analyses were performed using SPSS version 25 with the exception of the Bayes factors which were calculated using an online calculator (http://www.lifesci.sussex.ac.uk/home/Zoltan_Dienes/inference/Bayes.htm).

## 3 Results

Of the 6933 people who responded to the Smoking Toolkit Study survey between April and July 2008, 1439 reported smoking in the past year or currently using e-cigarettes and formed our analytic sample. Of these respondents, 1320 (91.7%) were past-year smokers, 1240 (86.2%) were current smokers, and 394 (27.4%) were e-cigarette users (of whom 150 (38.1%) were exclusive users and 244 (61.9%) were dual users of e-cigarettes and combustible tobacco) at the time of the survey. Sample characteristics are summarised in Table 1.

**Table 1:** Sample characteristics.

	Past-year smokers	Current smokers	Current e-cigarette users
	(*n* = 1320)	(*n* = 1240)	(*n* = 394)
Sex			
Men	51.7 (682)	51.8 (642)	51.8 (204)
Women	48.3 (638)	48.2 (598)	48.2 (190)

Age (years)			
16–24	16.7 (220)	16.7 (207)	11.7 (46)
25–34	20.9 (276)	20.4 (253)	21.3 (84)
35–44	16.1 (213)	16.4 (203)	20.8 (82)
45–54	18.2 (240)	17.9 (222)	21.8 (86)
55–64	13.6 (179)	13.9 (172)	13.5 (53)
≥65	14.5 (192)	14.8 (183)	10.9 (43)

Ethnicity			
White	88.6 (1164)	89.1 (1100)	91.6 (360)
Non-white	11.4 (150)	10.9 (134)	8.4 (33)

Social grade			
ABC1	40.6 (536)	40.6 (504)	44.7 (176)
C2DE	59.4 (784)	59.4 (736)	55.3 (218)

Tried to quit in past year			
No	70.0 (887)	73.1 (871)	48.3 (131)
Yes	30.0 (381)	26.9 (320)	51.7 (140)

Smoking status			
Current smoker	93.9 (1240)	100.0 (1240)	61.9 (244)
Quit in past year	6.1 (80)	–	7.9 (31)
Quit >1 year ago	–	–	25.4 (100)
Never smoked	–	–	4.8 (19)

Current e-cigarette use			
Yes	21.0 (277)	19.8 (246)	100.0 (394)
No	79.0 (1043)	80.2 (994)	–

Unweighted data. Data values are percentages (*n*).

**Fig. 1:**
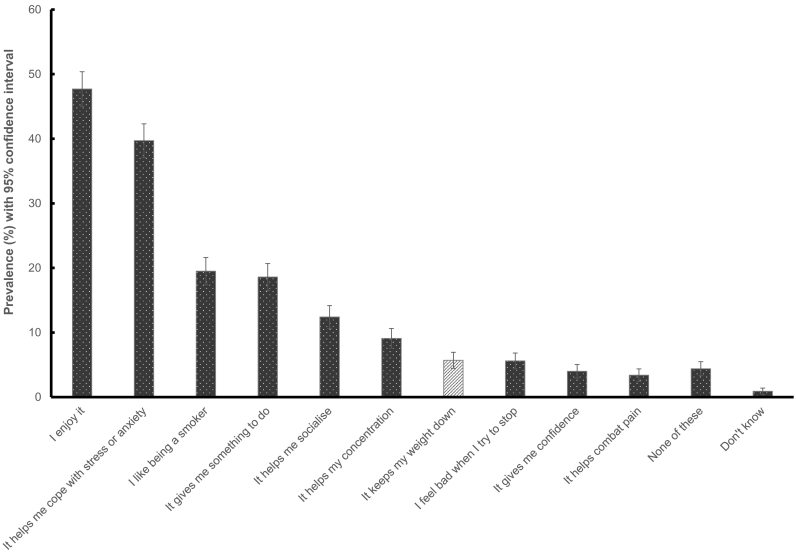
Prevalence of weight control compared with other motives for cigarette smoking among past-year smokers (*n* = 1320).

The prevalence of cigarette smoking for weight control among past-year smokers was 5.7% (95% CI 4.5% to 6.9%), making it one of the lesser reported motives for smoking (Fig. 1). The most commonly reported motives for cigarette smoking were enjoyment (47.7%), followed by relief from stress or anxiety (39.7%), liking being a smoker (19.5%) and as something to do (18.6%). The least commonly reported motive for cigarette smoking was pain relief (3.4%). Smoking for weight control was more commonly reported by respondents who were female, middle-aged and had quit smoking in the past year (Table 2). There were no significant differences by ethnicity, social grade or past-year quit attempts.

**Table 2:** Prevalence and adjusted odds of weight-related motives, usage patterns, beliefs and attitudes.

	Smoking for weight control^1^		Heard weight control claim^1^		Believe weight control claim^1^		Weight control would increase likelihood of e-cigarette use^2^		Weight control would increase likelihood of stopping smoking^2^	
	% [95% CI]	OR [95% CI]	% [95% CI]	OR [95% CI]	% [95% CI]	OR [95% CI]	% [95% CI]	OR [95% CI]	% [95% CI]	OR [95% CI]
Overall	5.7 [4.5–6.9]	–	8.8 [7.3–10.3]	–	6.4 [5.1–7.7]	–	13.4 [11.3–15.5]	–	13.1 [11.0–15.2]	–

Sex										
Men	3.6 [2.2–5.0]	1.00	10.6 [8.4–12.9]	1.00	8.5 [6.5–10.5]	1.00	11.8 [9.1–14.5]	1.00	11.8 [9.1–14.5]	1.00
Women	7.9 [5.8–10.0]	2.21 [1.34–3.63]⁎⁎	6.8 [4.8–8.8]	0.62 [0.42–0.93]⁎	4.0 [2.5–5.5]	0.44 [0.27–0.72]⁎⁎	15.3 [12.0–18.6]	1.33 [0.91–1.93]	14.8 [11.6–18.1]	1.25 [0.85–1.82]

Age (years)										
16–24	3.1 [0.8–5.4]	1.00	12.8 [8.4–17.2]	1.00	8.0 [4.5–11.5]	1.00	13.7 [8.5–18.9]	1.00	8.9 [4.6–13.2]	1.00
25–34	4.3 [2.1–6.5]	1.24 [0.49–3.11]	10.9 [7.5–14.3]	0.75 [0.44–1.28]	6.2 [3.6–8.8]	0.70 [0.36–1.36]	16.5 [11.8–21.2]	1.17 [0.66–2.05]	16.5 [11.8–21.2]	1.89 [0.996–3.59]
35–44	4.8 [2.1–7.5]	1.49 [0.58–3.86]	8.9 [5.4–12.4]	0.62 [0.35–1.13]	8.5 [5.0–12.0]	0.95 [0.49–1.86]	11.4 [6.8–16.0]	0.76 [0.40–1.44]	13.6 [8.7–18.6]	1.53 [0.77–3.05]
45–54	8.2 [4.8–11.6]	2.45 [1.01–5.93]⁎	6.9 [3.7–10.1]	0.49 [0.26–0.93]⁎	5.3 [2.5–8.1]	0.68 [0.32–1.42]	16.4 [10.9–22.0]	1.17 [0.63–2.15]	16.4 [10.9–22.0]	2.03 [1.03–4.00]⁎
55–64	9.6 [5.0–14.2]	2.97 [1.17–7.59]⁎	4.5 [1.3–7.8]	0.30 [0.13–0.72]⁎⁎	3.8 [0.8–6.8]	0.37 [0.13–1.05]	14.4 [8.1–20.7]	1.05 [0.53–2.08]	12.6 [6.6–18.6]	1.29 [0.59–2.84]
≥65	4.6 [1.3–7.9]	1.38 [0.47–4.03]	6.0 [2.2–9.8]	0.40 [0.18–0.89]⁎	5.3 [1.7–8.9]	0.71 [0.30–1.66]	4.2 [0.6–7.8]	0.26 [0.10–0.71]⁎⁎	7.6 [2.8–12.4]	0.80 [0.33–1.90]

Ethnicity										
White	5.9 [4.6–7.2]	1.00	9.0 [7.4–10.6]	1.00	6.3 [4.9–7.7]	1.00	13.8 [11.5–16.1]	1.00	13.4 [11.1–15.7]	1.00
Non-White	3.8 [0.8–6.8]	0.64 [0.27–1.53]	7.5 [3.4–11.6]	0.70 [0.37–1.32]	7.5 [3.4–11.6]	1.06 [0.55–2.06]	11.8 [6.0–17.6]	0.79 [0.43–1.45]	11.8 [6.0–17.6]	0.89 [0.48–1.63]

Social grade										
ABC1	5.1 [3.2–7.0]	1.00	10.3 [7.7–12.9]	1.00	5.5 [3.6–7.5]	1.00	11.2 [8.1–14.4]	1.00	10.1 [7.1–13.1]	1.00
C2DE	6.0 [4.4–6.7]	1.12 [0.69–1.84]	7.9 [6.1–9.7]	0.71 [0.48–1.05]	6.9 [5.2–8.6]	1.34 [0.84–2.14]	14.9 [12.1–17.7]	1.47 [0.99–2.18]	15.1 [12.3–17.9]	1.61 [1.07–2.42]⁎

Tried to quit in past year										
No	4.8 [3.4–6.8]	1.00	9.2 [7.3–11.1]	1.00	5.7 [4.2–7.2]	1.00	12.1 [9.8–14.4]	1.00	11.6 [9.3–13.9]	1.00
Yes	8.1 [5.4–10.8]	1.47 [0.88–2.47]	8.4 [5.6–11.2]	0.93 [0.60–1.45]	8.6 [5.8–11.4]	1.81 [1.13–2.91]⁎	20.7 [15.2–26.2]	1.92 [1.27–2.90]⁎⁎	20.2 [15.2–26.2]	1.94 [1.28–2.94]⁎⁎

Smoking status										
Current smoker	5.1 [3.9–6.3]	1.00	9.0 [7.4–10.6]	1.00	6.6 [5.2–8.0]	1.00	–	–	–	–
Quit in past year	13.8 [6.2–21.4]	2.58 [1.22–5.45]⁎	6.3 [1.0–11.6]	0.78 [0.30–2.00]	2.5 [0.0–5.9]	0.24 [0.05–1.08]	–	–	–	–

Data are weighted to match the English population on key sociodemographic variables. Odds ratios are mutually adjusted for all presented variables (sex, age, ethnicity, social grade, past-year quit attempts and current smoking status).

CI = confidence intervals; OR = odds ratio.

1 In past-year smokers.

2 In current smokers.

⁎ *p* < .05.

⁎⁎ *p* < .01.

**Fig. 2:**
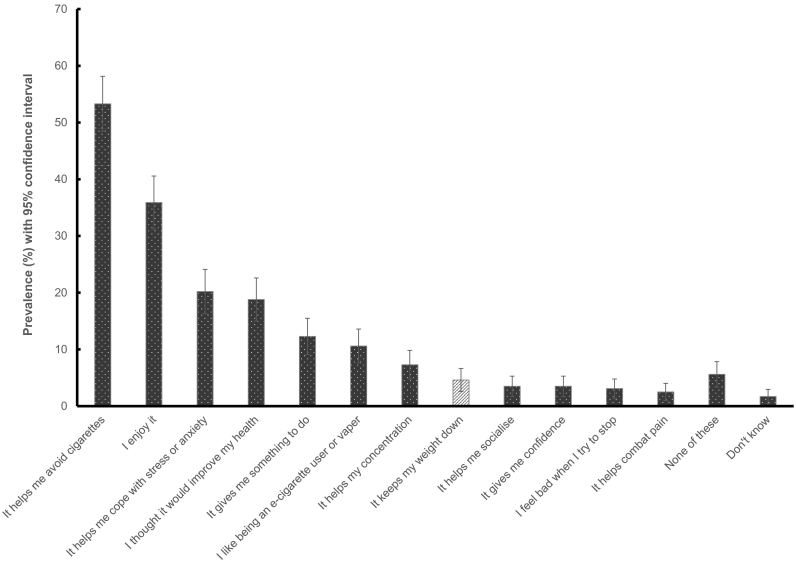
Prevalence of weight control compared with other motives for vaping among current e-cigarette users (*n* = 394).

Vaping for weight control was also reported infrequently, with 4.6% (95% CI 2.6% to 6.6%) of current e-cigarette users (4.3% (95% CI 1.2% to 7.4%) of exclusive users and 4.8% (95% CI 2.1% to 7.5%) of dual users) endorsing weight control as a motive for vaping. The most commonly reported motives for vaping were that it helps avoid cigarettes (53.3%), followed by enjoyment (35.9%), to relieve stress or anxiety (20.2%) and to improve health (18.8%) (Fig. 2). The least commonly reported motive for vaping was pain relief (2.5%). Due to the small number of vapers reporting weight control as a motive (unweighted *n* = 18), it was not possible to examine differences by sociodemographic or smoker characteristics.

**Fig. 3:**
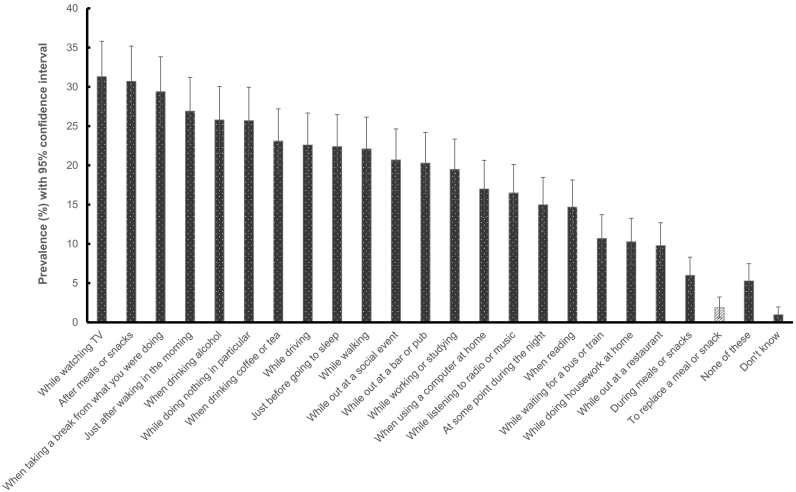
Prevalence of the use of e-cigarettes as a snack or meal replacement compared with other usage among current e-cigarette users (*n* = 394).

The prevalence of vaping to replace a meal or snack was also rare among current e-cigarette users (overall: 1.9%, 95% CI 0.6% to 3.2%; exclusive users: 1.5%, 95% CI 0% to 3.4%; dual users: 2.2%, 95% CI 0.4% to 4.1%). Of the 22 situations in which respondents were asked about their e-cigarette usage, vaping as a meal or snack replacement was the least commonly reported, while vaping while watching TV (31.3%) or after a meal or snack were the most commonly reported (30.7%) (Fig. 3). Among the small number of respondents who vaped to replace a meal or snack, 53.3% (95% CI 18.7% to 87.9%) reported doing so every day, 13.1% (95% CI 0% to 36.5%) did so less than every day but at least once a week, and the remaining 33.6% (95% CI 0.9% to 66.3%) did so less often than once a month. There was some inconsistent reporting, with 18.1% (95% CI 0% to 44.8%) of those who reported vaping to replace a meal or snack saying that they never did so when asked about frequency. Keeping hands busy (63.0%, 95% CI 29.5% to 96.5%), feeling in the mouth (58.8%, 95% CI 24.7% to 92.9%) and hand to mouth action (47.7%, 95% CI 13.1% to 82.3%) were rated as the most important factors in using an e-cigarette to replace a meal or snack. Density of vapour (30.2%, 95% CI 0% to 62.0%), nicotine concentration (16.6%, 95% CI 0% to 42.4%) and flavour (13.6%, 95% CI 0% to 37.4%) were also important to some respondents. Due to the small number reporting vaping to replace a meal or snack (unweighted *n* = 8), it was not possible to examine differences by sociodemographic or smoker characteristics.

In the full sample of past-year smokers, only 8.8% (95% CI 7.3% to 10.3%) had heard that using an e-cigarette or vaping device can help keep weight down. Just 6.4% (95% CI 5.1% to 7.7%) agreed that vaping can help keep weight down. Women were significantly less likely than men to have heard or to believe that vaping can help keep weight down (Table 2). Middle-aged respondents were less likely than younger respondents to have heard the claim, but belief that vaping can help keep weight down did not differ significantly by age (Table 2). Respondents who had tried to quit in the past year were no more likely to have heard the claim but were significantly more likely to believe it (Table 2). There were no significant differences by ethnicity, social grade or current smoking status.

Among current smokers who were not currently using e-cigarettes, insofar that vaping could help with weight control 13.4% (95% CI 11.3% to 15.5%) reported that they would be more likely to try e-cigarettes and 13.1% (95% CI 11.0% to 15.2%) more likely to stop smoking. In this event, the likelihood of trying e-cigarettes was lower among the oldest (≥65 years) compared with youngest respondents and higher among those who had tried to quit in the past year (Table 2). The likelihood of stopping smoking was higher among middle-aged (45–54 years) compared with the youngest respondents, higher among those who had tried to quit in the past year and higher among those from lower compared with higher social grades (Table 2). There were no other significant differences in likelihood of trying e-cigarettes or stopping smoking.

To interpret the non-significant results, Bayes factors were calculated to assess how far the results pointed to the null vs. the experimental hypothesis. Based on expected effect sizes of 2.0 and 0.5, the majority of Bayes factors indicated that data were insensitive or provided moderate to strong evidence for the null hypothesis (BF range 0.05–1.40) (Supplementary Table).

Sensitivity analyses performed on unweighted data revealed no notable differences in results (data not shown).

## 4 Discussion

These analyses show that one in 22 e-cigarette users in England vape in order to control their weight and one in 50 vape to replace meals or snacks. While the majority of individuals who had smoked in the past year had neither heard nor believed that vaping could help control weight, around one in eight current smokers said they would be more likely to try e-cigarettes or quit smoking completely if vaping could help to control their weight.

This is the first study, to our knowledge, that has explored the use of e-cigarettes for weight control in England. We observed substantially lower prevalence of vaping for weight control (4.6%) than was previously reported in a US survey (13.5%) (Morean & Wedel, 2017). Differences may relate to differences in study design: the present study was representative of the adult population in England, whereas the previous study used convenience sampling and required respondents to be currently trying to lose or maintain weight. While vaping after a meal or snack was commonly reported by e-cigarette users, the proportion who reported vaping to replace a meal or snack was low (1.9%). Although flavours might provide a direct alternative to food (Glover et al., 2017), few respondents in our study who vaped to replace food rated flavour as an important motive. Rather, approximately 6 out of 10 tended to report the physical and behavioural aspects of e-cigarette use (mouth feel, hand to mouth action, and keeping hands busy) as important. Performing ritualistic behaviours associated with vaping – for example, mixing the e-liquids, putting together and filling the device, and the act of vaping itself – may, like smoking, be used as a substitute for eating (Glover et al., 2017). Some respondents also reported that nicotine concentration and density of vapour were important, which may relate to the appetite-suppressing effects of nicotine (Jo, Talmage, & Role, 2002).

Only 9% of people who smoked in the previous year were aware that vaping may aid weight control. One reason why few people were aware could be that the rising prevalence of e-cigarette use in England has occurred in the context of an obesity epidemic. Approximately 26% of men and 27% of women in England are obese, and a further 40% of men and 30% of women are overweight (Health Survey for England, 2017). While there is not necessarily a causal link between rising levels of obesity and concurrent increase in rates of smoking cessation, it follows that a substantial proportion of e-cigarette users carry excess weight, which may undermine belief in claims linking vaping to weight control. If respondents had used e-cigarettes themselves and not noticed any co-occurring weight loss, this may also have contributed to doubts about the existence of link between vaping and weight control. Another reason that the majority of past-year smokers were unaware that vaping may aid weight control is because it has not been proven to do so, although a wider literature indicates that using alternative nicotine to support cessation inhibits associated weight gain (Farley et al., 2012).

The potential impact of finding and communicating evidence that vaping reduces weight gain on smoking cessation appeared modest: should e-cigarettes prove able to prevent weight gain, only 13.4% of current smokers said they would be more likely to try e-cigarettes and 13.1% said they would be more likely to stop smoking. However, these percentages should be interpreted in the light of several considerations. First, the prevalence of cigarette smoking for weight control among past-year smokers in our sample was just 5.7%, so e-cigarettes for weight control may appeal beyond those people who are smoking to control their weight to quit. Secondly, it is likely that intentions to quit if vaping could help control weight would be higher among the priority group of smokers who are overweight or obese. We did not have data on weight status but it is likely that at least a third of our general population sample would have been of a healthy weight (Statistics on Obesity, 2018). It is noteworthy that smokers who were middle-aged and from lower social grades – groups with higher than average prevalence of obesity (McLaren, 2007; Wang, McPherson, Marsh, Gortmaker, & Brown, 2011) – were significantly more likely to say they would stop smoking if vaping could help keep their weight down. Thirdly, it is not clear whether smokers who indicated that they would be more likely to stop smoking would switch to e-cigarettes to keep their weight down or quit using nicotine altogether, although the former appears more plausible in the UK context. Finally, the impact of a potential benefit of vaping for weight control would likely be higher among smokers with motivation to quit. We found that smokers who had made a serious quit attempt in the past year were significantly more likely than those who had not attempted to quit to say they would stop smoking if vaping could help keep their weight down.

The present results suggest that should an association between vaping and weight control be demonstrated, it might be possible to increase quit attempts by communicating this to smokers. However, this would need to be broached carefully, in order not to make vaping attractive to non-smokers for the purpose of weight control. While e-cigarettes have been shown to be much less harmful than continuing to smoke, they are not entirely without risk (McNeill, Brose, Calder, Bauld, & Robson, 2018). Messaging regarding potential but unproven effects of vaping on weight control may encourage uptake among groups otherwise unlikely to consider using e-cigarettes, particularly those with high body image concerns (e.g. adolescents).

Strengths of this study include the representative sample and detailed assessment of motives, behaviours and attitudes relevant to vaping for weight control. However, there were a number of limitations. The measures used to assess attitudes towards and experiences of vaping for weight control were not validated. Data were not collected on smoking combustible cigarettes to replace a meal or snack, or whether respondents had ever heard or agreed with the claim that smoking helps to keep weight down, so it was not possible to compare vaping with smoking for these variables. There was some inconsistency in reporting of vaping to replace a meal or snack, with 18.1% of those who reported doing so saying they never did so when pressed on frequency. Lack of attention or confusion are possible explanations, but it is also possible that those who vape to replace a meal or snack very infrequently selected ‘never’ when asked about frequency as they felt it better described their actions than ‘less than once a month’. Although the question asked about situations in which the respondents ‘regularly’ vaped, if the interviewer prompted ‘all that apply’, it is possible that responses were more likely ever use. The very low prevalence of vaping for weight control and vaping to replace a meal or snack meant we were unable to assess the correlation of these variables with socio-demographic and smoking characteristics. While we report descriptive data on frequency of using e-cigarettes to replace a meal or snack and important factors in doing so, confidence intervals for estimates of prevalence are very wide. Further research is required to validate these results and establish the extent to which these behaviours differ by sociodemographic and smoker characteristics. Sensitivity analyses using Bayes factors indicated that the data were insensitive to detect differences in many of the associations with awareness of the claim that vaping may aid weight control and belief and likelihood of trying e-cigarettes or quitting smoking insofar that claim was true, so it is possible that there are additional differences across sociodemographic groups that were not detected in this study.

In conclusion, only a small proportion of e-cigarette users in England report vaping to control their weight. Awareness among current smokers and recent ex-smokers of a potential link between vaping and weight control is currently low. However, if evidence that vaping could help users to control their weight during a quit attempt could be identified and communicated to smokers, they may be more inclined both to try e-cigarettes and to quit smoking.

## Role of funding sources

This work was supported by a TAG Cancer Research UK grant (grant number C22086/A24778). Wider data collection for the Smoking Toolkit Study and SEJ and JB's salaries were also funded by Cancer Research UK (grant number C1417/A22962). PA is an NIHR senior investigator and funded by NIHR Oxford Biomedical Research Centre and CLAHRC. The funders played no role in study design, collection, analysis, or interpretation of data, writing the manuscript, and the decision to submit the manuscript for publication.

## Contributors

JB, PA, FD and LB designed the study and acquired funding. SEJ conducted the statistical analyses and wrote the first draft of the manuscript. All authors contributed to and have approved the final manuscript.

## Conflict of interest

JB has received unrestricted research funding relating to smoking cessation from Pfizer, who manufacture smoking cessation medications. RW undertakes research and consultancy for and receives travel funds and hospitality from manufacturers of smoking cessation medications (Pfizer, GlaxoSmithKline and Johnson and Johnson). All authors declare no financial links with tobacco companies or e-cigarette manufacturers or their representatives.

## Ethical approval

Ethical approval for the STS was granted originally by the UCL Ethics Committee (ID 0498/001). The data are not collected by UCL and are anonymized when received by UCL.

## Appendix A Supplementary data

Supplementary material Image 1

## References

[bb0005] Aubin H.-J., Farley A., Lycett D., Lahmek P., Aveyard P. (2012 Jul 10). Weight gain in smokers after quitting cigarettes: Meta-analysis. BMJ.

[bb0010] Bauld L., Graham H., Sinclair L., Flemming K., Naughton F., Ford A. (2017). Barriers to and facilitators of smoking cessation in pregnancy and following childbirth: Literature review and qualitative study. Health Technology Assessment.

[bb0015] Bennett B., Pokhrel P., Bennett B.L., Pokhrel P. (2018 May 28). Weight concerns and use of cigarettes and e-cigarettes among young adults. International Journal of Environmental Research and Public Health.

[bb0020] Boles S.M., Johnson P.B. (2001). Gender, weight concerns, and adolescent smoking. Journal of Addictive Diseases.

[bb0025] Borrelli B., Mermelstein R. (1998 Oct). The role of weight concern and self-efficacy in smoking cessation and weight gain among smokers in a clinic-based cessation program. Addictive Behaviors.

[bb0030] Brown J., West R., Beard E., Michie S., Shahab L., McNeill A. (2014 Jun 1). Prevalence and characteristics of e-cigarette users in Great Britain: Findings from a general population survey of smokers. Addictive Behaviors.

[bb0035] Camp D.E., Klesges R.C., Relyea G. (1993 Jan). The relationship between body weight concerns and adolescent smoking. Health Psychology Official Journal Division of Health Psychology: Americal Psychological Association.

[bb0040] Courtemanche C.J., Pinkston J.C., Ruhm C.J., Wehby G.L. (2016 Apr 1). Can changing economic factors explain the rise in obesity?. Southern Economic Journal.

[bb0045] Dare S., Mackay D.F., Pell J.P. (2015 Apr 17). Relationship between smoking and obesity: A cross-sectional study of 499,504 middle-aged adults in the UK general population. PLoS One.

[bb0050] Dienes Z. (2014 Jul 29). Using Bayes to get the most out of non-significant results. Frontiers in Psychology.

[bb0055] Farley A.C., Hajek P., Lycett D., Aveyard P. (2012 Jan 18). Interventions for preventing weight gain after smoking cessation. Cochrane Database of Systematic Reviews.

[bb0060] Fidler J.A., Shahab L., West O., Jarvis M.J., McEwen A., Stapleton J.A. (2011 Jun 18). “The smoking toolkit study”: A national study of smoking and smoking cessation in England. BMC Public Health.

[bb0065] Fidler J.A., West R. (2009 Oct 1). Self-perceived smoking motives and their correlates in a general population sample. Nicotine & Tobacco Research.

[bb0070] Flemming K., McCaughan D., Angus K., Graham H. (2015 Jun). Qualitative systematic review: Barriers and facilitators to smoking cessation experienced by women in pregnancy and following childbirth. Journal of Advanced Nursing.

[bb0075] French S.A., Perry C.L., Leon G.R., Fulkerson J.A. (1994 Nov). Weight concerns, dieting behavior, and smoking initiation among adolescents: A prospective study. American Journal of Public Health.

[bb0080] Glover M., Breier B.H., Bauld L. (2017 Nov 7). Could vaping be a new weapon in the battle of the bulge?. Nicotine & Tobacco Research.

[bb0085] Hall S.M., Tunstall C.D., Vila K.L., Duffy J. (1992 Jun). Weight gain prevention and smoking cessation: Cautionary findings. American Journal of Public Health.

[bb0090] Health Survey for England (2017). Key findings: Adult overweight and obesity [internet]. http://healthsurvey.hscic.gov.uk/support-guidance/public-health/health-survey-for-england-2016/key-findings.aspx.

[bb0095] Jeffreys H. (1961). The theory of probability.

[bb0100] Jo Y.-H., Talmage D.A., Role L.W. (2002 Dec 1). Nicotinic receptor-mediated effects on appetite and food intake. Journal of Neurobiology.

[bb0105] Levy D.T., Yuan Z., Li Y. (2017 Oct). The prevalence and characteristics of e-cigarette users in the U.S. International Journal of Environmental Research and Public Health.

[bb0110] Lycett D., Munafò M., Johnstone E., Murphy M., Aveyard P. (2011 Jan). Associations between weight change over 8 years and baseline body mass index in a cohort of continuing and quitting smokers. Addiction.

[bb0115] Martínez J.A., Kearney J.M., Kafatos A., Paquet S., Martínez-Gonzélez M.A. (1999 Jan). Variables independently associated with self-reported obesity in the European Union. Public Health Nutrition.

[bb0120] McLaren L. (2007 Jan 1). Socioeconomic status and obesity. Epidemiologic Reviews.

[bb0125] McNeill A., Brose L.S., Calder R., Bauld L., Robson D. (2018). Evidence review of e-cigarettes and heated tobacco products 2018. Report on Common Public Health England.

[bb0130] Morean M.E., Wedel A.V. (2017 Mar). Vaping to lose weight: Predictors of adult e-cigarette use for weight loss or control. Addictive Behaviors.

[bb0135] Statistics on Obesity (2018). Physical activity and diet - England, 2018 [PAS] - NHS digital. https://digital.nhs.uk/data-and-information/publications/statistical/statistics-on-obesity-physical-activity-and-diet/statistics-on-obesity-physical-activity-and-diet-england-2018.

[bb0140] Vardavas C.I., Filippidis F.T., Agaku I.T. (2015 Sep 1). Determinants and prevalence of e-cigarette use throughout the European Union: A secondary analysis of 26 566 youth and adults from 27 countries. Tobacco Control.

[bb0145] Voorhees C.C., Schreiber G.B., Schumann B.C., Biro F., Crawford P.B. (2002). Early predictors of daily smoking in young women: The National Heart, Lung, and Blood Institute Growth and Health Study. Preventive Medicine an International Journal Devoted to Practice and Theory.

[bb0150] Wang Y.C., McPherson K., Marsh T., Gortmaker S.L., Brown M. (2011). Health and economic burden of the projected obesity trends in the USA and the UK. The Lancet.

[bb0155] Wardle J., Griffith J. (2001 Mar 1). Socioeconomic status and weight control practices in British adults. Journal of Epidemiology and Community Health.

[bb0160] Wee C.C., Rigotti N.A., Davis R.B., Phillips R.S. (2001 Feb 26). Relationship between smoking and weight control efforts among adults in the United States. Archives of Internal Medicine.

[bb0165] West R. (2015). Using Bayesian analysis for hypothesis testing in addiction science. Addiction.

[bb0170] West R., Brown J. Electronic cigarettes in England - Latest trends. http://www.smokinginengland.info/latest-statistics/.

[bb0175] West R., Hajek P., Nilsson F., Foulds J., May S., Meadows A. (2001 Jan 1). Individual differences in preferences for and responses to four nicotine replacement products. Psychopharmacology.

[bb0180] West R., Shahab L., Brown J. (2016 Jun 1). Estimating the population impact of e-cigarettes on smoking cessation in England. Addiction..

[bb0185] West R.J., Russell M.A., Jarvis M.J., Feyerabend C. (1984). Does switching to an ultra-low nicotine cigarette induce nicotine withdrawal effects?. Psychopharmacology.

[bb0190] Winsløw U.C., Rode L., Nordestgaard B.G. (2015 Apr). High tobacco consumption lowers body weight: A Mendelian randomization study of the Copenhagen General Population Study. Journal of Epidemiology.

[bb0195] Yaemsiri S., Slining M.M., Agarwal S.K. (2011 Aug 1). Perceived weight status, overweight diagnosis, and weight control among US adults: The NHANES 2003–2008 Study. International Journal of Obesity.

