# Supplementary material for: Vaping for weight control: A cross-sectional population study in England
Source: Addict Behav. 2019 Aug;95:211–9. doi: 10.1016/j.addbeh.2019.04.007 (PMC6555398; doi:10.1016/j.addbeh.2019.04.007)
Supplement: Supplementary file 1 — Supplementary material [file mmc1.pdf]

Supplementary Table Bayes factors for non-significant results

	BF 2.0	Interpretation of BF based on 2.0	BF 0.5	Interpretation of BF based on 0.5
**Smoking for weight control**				
Age 25-34	0.84	Data are insensitive	0.40	Data are insensitive
Age 35-44	1.00	Data are insensitive	0.26	Moderate evidence for the null hypothesis
Age 65+	0.95	Data are insensitive	0.39	Data are insensitive
Non-white	0.19	Moderate evidence for the null hypothesis	1.04	Data are insensitive
C2DE	0.53	Data are insensitive	0.22	Moderate evidence for the null hypothesis
Tried to quit in past year	1.11	Data are insensitive	0.09	Strong evidence for the null hypothesis
**Heard weight control claim**				
Age 25-34	0.13	Moderate evidence for the null hypothesis	0.90	Data are insensitive
Age 35-44	0.08	Strong evidence for the null hypothesis	1.20	Data are insensitive
Non-white	0.14	Moderate evidence for the null hypothesis	0.99	Data are insensitive
C2DE	0.06	Strong evidence for the null hypothesis	1.21	Data are insensitive
Tried to quit in past year	0.24	Moderate evidence for the null hypothesis	0.42	Data are insensitive
Quit in past year	0.82	Data are insensitive	0.88	Data are insensitive
**Believe weight control claim**				
Age 25-34	0.16	Moderate evidence for the null hypothesis	0.99	Data are insensitive
Age 35-44	0.41	Data are insensitive	0.53	Data are insensitive
Age 45-54	0.17	Moderate evidence for the null hypothesis	1.01	Data are insensitive
Age 55-64	0.06	Strong evidence for the null hypothesis	1.11	Data are insensitive
Age 65+	0.25	Moderate evidence for the null hypothesis	0.96	Data are insensitive
Non-white	0.54	Data are insensitive	0.39	Data are insensitive
C2DE	0.93	Data are insensitive	0.11	Moderate evidence for the null hypothesis
Quit in past year	0.05	Strong evidence for the null hypothesis	1.02	Data are insensitive
**Weight control would increase likelihood of e-cigarette use**				
Female	1.01	Data are insensitive	0.07	Strong evidence for the null hypothesis
Age 25-34	0.64	Data are insensitive	0.24	Moderate evidence for the null hypothesis
Age 35-44	0.19	Moderate evidence for the null hypothesis	0.87	Data are insensitive
Age 45-54	0.66	Data are insensitive	0.27	Moderate evidence for the null hypothesis
Age 55-64	0.54	Data are insensitive	0.42	Data are insensitive
Non-white	0.20	Moderate evidence for the null hypothesis	0.80	Data are insensitive
C2DE	1.40	Data are insensitive	0.05	Strong evidence for the null hypothesis
**Weight control would increase likelihood of stopping smoking**				
Female	0.77	Data are insensitive	0.10	Moderate evidence for the null hypothesis
Age 25-34	1.33	Data are insensitive	0.05	Strong evidence for the null hypothesis
Age 35-44	1.07	Data are insensitive	0.13	Moderate evidence for the null hypothesis
Age 55-64	0.85	Data are insensitive	0.29	Moderate evidence for the null hypothesis
Age 65+	0.35	Data are insensitive	0.85	Data are insensitive
Non-white	0.29	Moderate evidence for the null hypothesis	0.60	Data are insensitive

BF 2.0 = Bayes factor based on an expected effect size of OR=2.0. BF 0.5 = Bayes factor based on an expected effect size of OR=0.5. Bayes factors between 1/30 and 1/10 indicate strong evidence for the null hypothesis, between 1/10 and 1/3 indicate moderate evidence for the null hypothesis, and between 1/3 and 3 indicate that the data are insensitive (based on Jeffreys' Bayes factor cut-offs (21,24)).
